# Establishing the reference intervals for subpopulations of T lymphocytes, B lymphocytes, and natural killer cells: insight from the Brazilian Longitudinal Study of Adult Health (ELSA-Brasil)

**DOI:** 10.1590/1414-431X2025e14956

**Published:** 2025-10-17

**Authors:** I.C.S. Dias, C.B. Maluf, S.G. Xavier, L. Giatti, S.M. Barreto, P.G. Vidigal

**Affiliations:** 1 Programa de Pós-Graduação em Patologia, Universidade Federal de Minas Gerais, Belo Horizonte, MG, Brasil; 2 Departamento de Propedêutica Complementar, Faculdade de Medicina, Universidade Federal de Minas Gerais, Belo Horizonte, MG, Brasil; 3 Programa de Pós-Graduação em Saúde Pública, Faculdade de Medicina e EBSERH/Hospital de Clínicas, Universidade Federal de Minas Gerais, Belo Horizonte, MG, Brasil

**Keywords:** Reference value, Lymphocyte subpopulations, Flow cytometry

## Abstract

The aim of this study was to establish reference intervals for lymphocyte subpopulations in the peripheral blood of Brazilian adults, and to assess potential variations by gender and age groups. The study assessed 351 healthy participants of the Brazilian Longitudinal Study of Adult Health (ELSA-Brasil). Lymphocyte subpopulations were analyzed by dual platform using automated hematological analyzer Sysmex XN-3000 and the four-color flow cytometer on the FACSCalibur. Reference intervals were established using the 2.5th and 97.5th percentiles. Z-score was employed to ascertain the need for distinct reference intervals across gender and age groups. The Mann-Whitney test, with a significance level set at P<0.05, was conducted to identify differences among population groups. The absolute and relative reference intervals were: total lymphocytes: 1.0-2.9 (×10^3^/µL); CD3+: 721.0-2311.5 cells/μL, 59.1-84.5%; CD4+: 421.4-1523.8 cells/μL, 32.5-61.6%; CD8+: 175.3-879.8 cells/μL, 12.3-39.1%; CD4+CD8+: 0.8-4.1 cells/μL; CD19+: 85.2-501.6 cells/μL, 5.6-21.0%; NK: 83.9-444.4 cells/μL, 4.3-23.5%. Significant gender and age differences were observed in both the relative and absolute values of most lymphocyte subpopulations. There are variations in lymphocyte subsets across the global population, underscoring the need to establish tailored reference intervals for distinct populations, particularly for Helper T lymphocytes, B lymphocytes, and NK cells.

## Introduction

The analysis of lymphocyte subsets is pivotal in addressing primary immunodeficiencies, a set of genetically diverse diseases that impact various components of the innate and adaptive immunity, including neutrophils, macrophages, dendritic cells, complement system proteins, natural killer (NK) cells, as well as B and T lymphocytes. It is also beneficial in managing acquired immunodeficiencies, particularly in the initial laboratory assessment of Human Immunodeficiency Virus (HIV)-infected patients. This helps to evaluate the overall health status and the effectiveness of antiretroviral therapy (1). Moreover, it holds prognostic significance in other diseases, such as coronavirus disease (COVID-19), sepsis, lymphoproliferative disorders, and cancer (2-6). Laboratory test results are frequently compared to reference values or intervals for clinical interpretation, decision-making, and patient monitoring. Essential factors in determining an appropriate reference interval include population characteristics and control of pre-analytical and analytical interferences. The reference value for a specific analyte within a population can be determined from measurements taken in a relatively small but representative group of individuals (7).

Few Brazilian studies have specifically evaluated lymphocyte and NK cell subpopulations in adults. Despite these Brazilian studies, most reference ranges for lymphocyte subpopulations used in Brazil are based on data from other countries (8). Therefore, the aim of this study was to establish reference intervals for lymphocyte subpopulations in the peripheral blood of Brazilian adults and to assess potential variations by gender and age groups.

## Material and Methods

### Study population

The study population comes from the Brazilian Longitudinal Study of Adult Health (ELSA-Brasil), a prospective multicenter and multiethnic study of public civil servants from teaching and research institutions located in six Brazilian states (Minas Gerais, São Paulo, Rio de Janeiro, Espírito Santo, Bahia, and Rio Grande do Sul). This cross-sectional study was conducted with a convenience sample of 556 participants from the third study visit of ELSA-Brasil, who underwent data collection at the Minas Gerais Investigation Center. The baseline visit for ELSA-Brasil took place between 2008 and 2010, the second study visit occurred from 2012 to 2014, and the third study visit spanned from 2016 to 2018. Further details on the study design and cohort profile can be found elsewhere (9).

All 556 participants, aged between 42 and 82 years, underwent blood collection to determine lymphocytes and their respective subpopulations. Demographic variables (gender, age, race), smoking, and alcohol consumption data were retrieved from the ELSA-Brasil Project database.

Age groups were defined based on the criteria set by the World Health Organization (WHO), categorizing adults as individuals under 60 years old and elderly individuals aged 60 years or older (10).

The exclusion criteria were: 1) self-reported neoplasia documented at the study baseline; 2) history of bariatric surgery; 3) self-report of recent infectious conditions or any health issues within the past 12 h; 4) self-rated health status as ‘bad’ or ‘very bad’; 5) self-reported autoimmune diseases (such as arthritis, lupus, colitis, and psoriasis) at the third study visit; 6) use of immunosuppressants; 7) use of antivirals; 8) use of antibiotics; 9) use of nonsteroidal anti-inflammatory drugs; 10) use of corticosteroids; and 11) C-reactive protein levels ≥10 mg/L at the third study visit.

In a preliminary analysis using smoking, excessive drinking, hypertension, and diabetes as exclusion factors, no significant difference was observed in the reference range defined for these cells (data not shown). Consequently, these individuals were not excluded from the study.

### Determination of lymphocyte subpopulations

Between December 2017 and April 2018, 5 mL of venous blood was collected in ethylenediaminetetraacetic acid (EDTA) tubes. To minimize circadian variability, venous blood was obtained at the same time of the day, between 7:30 and 9:30. The total lymphocyte count of all the subjects was within the reference range (1.000-2.900/μL). Samples were stored at room temperature until staining.

Lymphocyte subpopulations were analyzed by dual platform using the automated hematological analyzer Sysmex XN-3000 (Sysmex Corporation, Japan) and the four-color flow cytometer FACSCalibur (BD Biosciences, USA). Total lymphocyte count was also measured with Sysmex XN-3000.

Four-color combinations of the following monoclonal antibodies were used: CD45-PerCP/CD3-FITC/CD4-APC/CD8-PE/CD19-APC, and CD16+CD56-PE (BD Multitest). EDTA blood (50 µL) was added to an aliquot of monoclonal antibody as suggested by the manufacturer and incubated for 15 min protected from light at room temperature. After incubation, erythrocytes were lysed with BD FACS Lysing Solution (BD Biosciences). Samples were then acquired on the flow cytometer.

Staining was always undertaken within 4 h of sampling. Four-color-flow cytometry analysis was performed on the FACSCalibur™ flow cytometer (BD Biosciences). Acquisition was run until 20,000 events were detected. Data analyses were made with MultiSET software.

Using the appropriate “gating” approach, the relative frequencies were determined for T-cells (CD3+), helper T cells (CD3+CD4+), cytolytic T-cells (CD3+CD8+), B-cells (CD3-CD19+), and NK-cells (CD3-CD16+/56+). Since we worked with a dual platform, the absolute frequencies of the cell's subsets were calculated based on the relative frequencies of the total lymphocytes.

Calibration and compensation of the flow cytometer were performed daily using the BD Calibrite™ reagent, as indicated on the package insert. During the calibration process, time delay verification, sensitivity testing and voltage adjustment of the photomultiplier (PMTs) were carried out, and the report was stored in the instruments. All the calibrators were stored in cool place at a controlled temperature.

The total lymphocyte count and relative frequency of lymphocyte subpopulations was measured in the laboratory of the University Hospital of the Universidade Federal de Minas Gerais (UFMG), which participates in the following external quality control programs: Excellence Program for Medical Laboratories (PELM) of the Brazilian Society of Clinical Pathology and Laboratory Medicine (SBPC/ML) and United Kingdom National External Quality Assessment Service (UK NEQAS) - Leucocyte Immunophenotyping.

### Statistical analysis

The distributions of study variables were summarized using frequency, mean, standard deviation, and median. To evaluate potential deviations from normality, Skewness, Kurtosis, and Kolmogorov-Smirnov tests were employed. Differences between subgroups were examined using the Mann-Whitney U test, with a significance level of P<0.05.

Outlier identification within each subgroup was conducted using the Dixon and Reed method, commonly referred to as the D/R ratio. Extreme values were omitted from the distribution if D/R≥1/3 (7,11).

Reference intervals were calculated using a non-parametric method in accordance with the Clinical & Laboratory Standards Institute (CLSI) guidelines. The 2.5th and 97.5th percentiles were utilized as the lower and upper reference limits, respectively, providing a 95% confidence interval (7).

The necessity of recommending specific reference intervals for lymphocyte subpopulations based on age and gender was assessed using the Z-score. The Z-score was calculated from the means and standard deviations of each group (11,12), as demonstrated in Equation 1, where SD is standard deviation and N is the number of individuals of each group. Equation 1. Z-Score Calculation:

$$Z=\frac{Mean\;1-Mean\;2}{{\left(SD1/N1+SD2/N2\right)}^{1/2}}$$ (Eq. 1)

The calculated Z-score was then compared with the critical Z-score (Z*), as demonstrated in Equation 2, where N is the number of individuals in each group and 240 is the sum of at least 120 individuals that must be in each group for partitioning. Equation 2. Calculation of the critical Z-score (Z*):

$$Z^{*}=3{\left[(N1+N2)/240\right]}^{1/2}$$ (Eq. 2)

Specific reference intervals are recommended if any of the following conditions are met: 1) the calculated Z-score exceeds the critical Z-score value (Z>Z*); 2) statistical differences exist between the parameters and the standard deviation of each subgroup. This criterion is met when the larger standard deviation exceeds the smaller one by 1.5 times, or when the ratio [larger standard deviation / (larger standard deviation - smaller standard deviation)] is less than 3 (7,13).

### Ethical aspects

Biological samples were collected from ELSA-Brasil project participants after obtaining free and informed consent. The ELSA-Brasil project was approved by the Ethics Committee (186/06) of Universidade Federal de Minas Gerais by the participants of the third wave of assessment (1.901.617).

## Results

### Sociodemographic and clinical data

**Figure 1:**
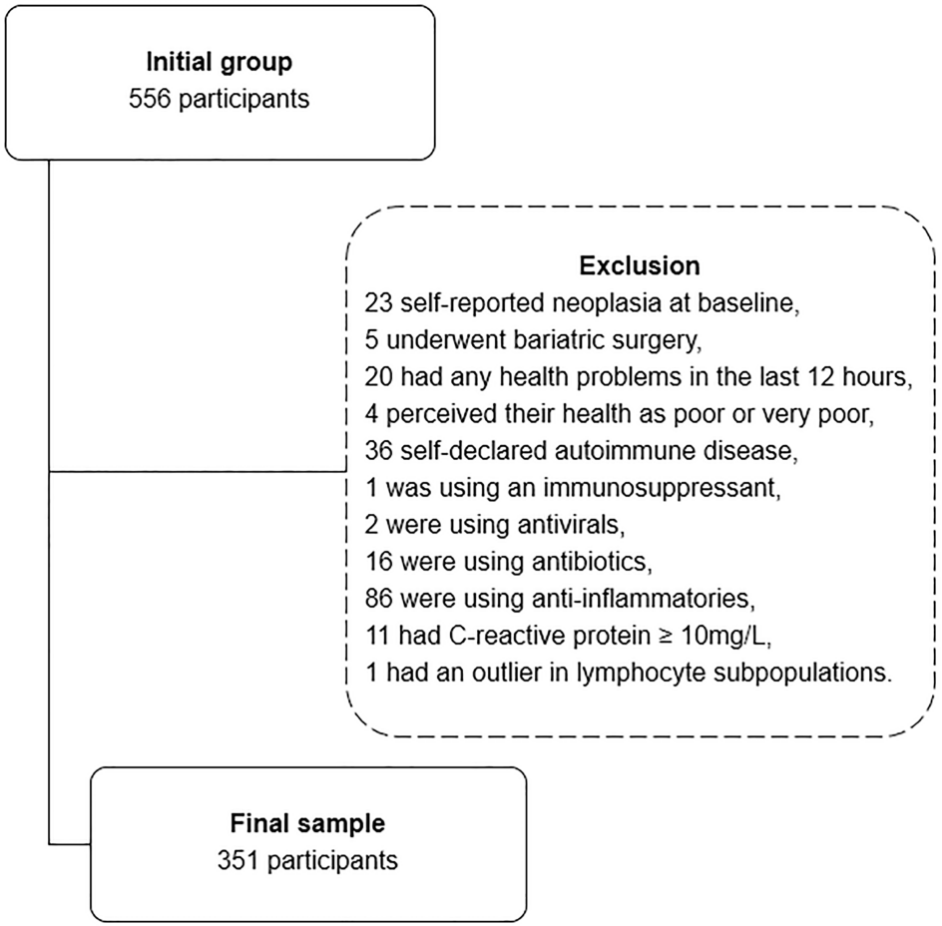
Participant selection based on exclusion criteria.

Based on the exclusion criteria, 205 of the initial 556 participants were excluded, as shown in Figure 1, resulting in 351 participants for determining the reference intervals.

The sociodemographic characteristics of this population are shown in Table 1. The reference population exhibited a slight male predominance (50.1%) and had an average age of 58.6 years, with 54.4% being adults. The majority was white (52.9%) and had completed higher education or postgraduate studies (74.5%).

**Table 1:** Sociodemographic characteristics of the reference population.

Characteristic	Participants (n=351)
Gender	
Male, n (%)	176 (50.1)
Female, n (%)	175 (49.8)
Mean age±SD (years)	58.6±8.1
Age range (years)	42-82
Age groups	
Adult <60 years, n (%)	191 (54.4)
Elderly ≥60 years, n (%)	160 (45.5)
Race	
White, n (%)	186 (52.9)
Black and Brown, n (%)	154 (43.8)
Other^a^, n (%)	10 (2.8)
Education	
Elementary, n (%)	16 (4.5)
High school, n (%)	73 (20.8)
Higher education, n (%)	82 (23.3)
Postgraduate, n (%)	180 (51.2)

^a^Other (Asian and Indigenous).

### Comparison between groups

The absolute and relative values of nearly all lymphocyte subtypes were significantly higher in women, except for NK cells, which were significantly more numerous in men (P=0.0001) (Table 2).

**Table 2:** Total lymphocytes and lymphocyte subpopulation count by gender.

Parameter	Median (P25- P75)		P value
	Male (n=176)	Female (n=175)	
Total lymphocytes (×10^3^/µL)	1.7 (1.4-2.1)	1.8 (1.5-2.1)	0.0999
T lymphocytes (CD3+)			
Absolute number (cells/µL)	1276.7 (1039.9-1558.6)	1388.8 (1119.9-1692.3)	**0.0211**
Relative number (%)	72.3 (67.9-77.3)	74.5 (70.9-78.2)	**0.0136**
Helper T lymphocytes (CD3+/CD4+)			
Absolute number (cells/µL)	787.5 (626.7-992.6)	916.1 (740.5-1139.4)	**0.0002**
Relative number (%)	46 (40.3-50.2)	49.2 (43.8-54.7)	**0.0000**
Cytotoxic T lymphocytes (CD3+/CD8+)			
Absolute number (cells/µL)	443.0 (308.5-581.4)	435.5 (334.5-571.4)	0.8568
Relative number (%)	24.3 (18.7-30.9)	22.8 (18.8-28.1)	0.1245
CD4+/CD8+ ratio	1.8 (1.3-2.5)	2.1 (1.6-2.7)	**0.0048**
B lymphocytes (CD19+)*			
Absolute number (cells/µL)	197.6 (148.5-264.5)	235.7 (187.6-329.7)	**0.0000**
Relative number (%)	10.9 (8.4-14.3)	13.5 (10.42-16.2)	**0.0000**
Natural killer cells (NK)*			
Absolute number (cells/µL)	221.0 (159.5-304.2)	184.3 (144.7-245.0)	**0.0001**
Relative number (%)	12.2 (9.5-17.3)	9.9 (7.5-12.7)	**0.0000**

CD: cluster of differentiation. *The sum of observations may differ in some variables due to missing data. Values in bold indicate a significant difference (Mann-Whitney test).

Regarding age groups, the absolute number of T lymphocytes (P=0.0480), the absolute and relative numbers of cytotoxic T lymphocytes (P=0.065), and B lymphocytes (P=0.0004) were significantly higher in adults. Conversely, in the elderly group, the helper T lymphocyte/cytotoxic T lymphocytes ratio (P=0.0487) and NK cells were significantly higher (P=0.0311) (Table 3).

**Table 3:** Total lymphocytes and lymphocyte subpopulation count by age group.

Parameter	Median (P25-P75)		P value
	Adult (n=191)	Elderly (n=160)	
Total lymphocytes (×10^3^/µL)	1.8 (1.5-2.2)	1.7 (1.4-2.1)	0.0566
T lymphocytes (CD3+)			
Absolute number (cells/µL)	1344.6 (1114.5-1696.0)	1337.7 (1029.7-1579.6)	**0.0480**
Relative number (%)	74.5 (70.3-78.5)	73.4 (68.7-77.6)	0.2195
Helper T lymphocytes (CD3+/CD4+)			
Absolute number (cells/µL)	864.5 (690.7-1121.2)	804.1 (650.2-1065.1)	0.1711
Relative number (%)	47.2 (42.4-51.8)	47.7 (41.8-54.6)	0.5153
Cytotoxic T lymphocytes (CD3+/CD8+)			
Absolute number (cells/µL)	468.2 (337.8-589.1)	405.2 (298.5-538.0)	**0.0065**
Relative number (%)	24.49 (20.2-29.3)	22.5 (17.6-28.4)	**0.0217**
CD4+/CD8+ Ratio	1.8 (1.4-2.4)	2.1 (1.4-2.8)	**0.0487**
B Lymphocyte (CD19+)*			
Absolute number (cells/µL)	233.2 (178.0-317.1)	198.9 (146.0-263.5)	**0.0004**
Relative number (%)	12.9 (10.0-15.7)	11.0 (8.4-14.5)	**0.0008**
Natural killer cells (NK)*			
Absolute number (cells/µL)	190.1 (150.6-255.1)	213.6 (153.5-282.5)	**0.0311**
Relative number (%)	10.1 (7.8-13.5)	11.9 (9.2-16.6)	**0.0003**

CD: cluster of differentiation. *The sum of observations may differ in some variables due to missing data. Values in bold indicate a significant difference (Mann-Whitney test).

### Reference intervals

Table 4 presents the median and 2.5th and 97.5th percentiles representing the lower and upper limits of the reference intervals for lymphocyte subpopulations.

**Table 4:** Reference intervals for lymphocyte subpopulations for the study population.

Parameter	Reference intervals	Median
Total lymphocytes (×10^3^/µL)	1.0-2.9	1.8
T lymphocytes (CD3+)		
Absolute number (cells/µL)	721.0-2311.5	1339.6
Relative number (%)	59.1-84.5	73.6
Helper T lymphocytes (CD3+/CD4+)		
Absolute number (cells/µL)	421.4-1523.8	831.4
Relative number (%)	32.5-61.6	47.3
Cytotoxic T lymphocytes (CD3+/CD8+)		
Absolute number (cells/µL)	175.3-879.8	436.6
Relative number (%)	12.3-39.1	23.1
CD4+/CD8+ ratio	0.8-4.1	1.9
B lymphocytes (CD19+)*		
Absolute number (cells/µL)	85.2-501.6	215.2
Relative number (%)	5.68-21.0	12.1
Natural killer cells (NK)*		
Absolute number (cells/µL)	83.9-444.4	202.8
Relative number (%)	4.3-23.5	10.9

CD: cluster of differentiation. *The sum of observations may differ in some variables due to missing data.

### Reference intervals by gender

Table 5 presents the reference values for lymphocyte subpopulations according to gender. The Harris-Boyd Z-test results indicated the need for defining gender-specific reference intervals for the absolute and relative counts of helper T lymphocytes, B lymphocytes, and NK cells. The results for the other lymphocyte subpopulations did not indicate the same need.

**Table 5:** Reference intervals for lymphocyte subpopulations by gender.

Parameter	Reference intervals		Median	
	Male (n=176)	Female (n=175)	Male (n=176)	Female (n=175)
Total lymphocytes (×10^3^/µL)	1.0-2.8	1.1-3.0	1.7	1.8
T lymphocytes (CD3+)				
Absolute number (cells/µL)	710.1-2209.7	783.8-2324.3	1276.7	1388.8
Relative number (%)	57.7-87.9	62.4-84.5	72.3	74.5
Helper T lymphocytes (CD3+/CD4+)				
Absolute number (cells/µL)	403.6-1445.1	447.4-1645.4	787.5	916.1
Relative number (%)	28.9-60.7	35.9-62.0	46	49.2
Cytotoxic T lymphocytes (CD3+/CD8+)				
Absolute number (cells/µL)	175.5-932.2	175.3-851.3	443.0	435.5
Relative number (%)	12.5-41.4	12.0-36.2	24.35	22.8
CD4+/CD8+ Ratio	0.6-4.0	0.9-4.2	1.8	2.1
B lymphocytes (CD19+)*				
Absolute number (cells/µL)	71.6-458.6	87.2-547.9	197.6	235.7
Relative number (%)	5.3-20.1	5.9-22.1	10.9	13.5
Natural killer cells (NK)*				
Absolute number (cells/µL)	83.9-477.2	85.8-368.7	221.0	184.3
Relative number (%)	5.2-25.8	4.0-18.9	12.2	9.9

CD: cluster of differentiation. *The sum of observations may differ in some variables due to missing data.

### Reference intervals by age

Table 6 presents the reference intervals for lymphocyte subpopulations categorized by age. The Harris-Boyd Z-test results indicated that there was no need for age-specific reference intervals for adults and the elderly.

**Table 6:** Reference intervals for lymphocyte subpopulations by age.

Parameter	Reference Intervals		Median	
	Adult (n=191)	Elderly (n=160)	Adult (n=191)	Elderly (n=160)
Total lymphocytes (×10^3^/µL)	1.1-2.9	1.0-2.9	1.8	1.7
T lymphocytes (CD3+)				
Absolute number (cells/µL)	808.6-2280.4	651.7-2398.2	1344.6	1337.7
Relative number (%)	59.4-84.1	57.7-85.3	74.52	73.4
Helper T lymphocytes (CD3+/CD4+)				
Absolute number (cells/µL)	447.4-1521.1	375.9-1549.6	864.5	804.1
Relative number (%)	32.6-61.2	32.1-61.8	47.2	47.7
Cytotoxic T lymphocytes (CD3+/CD8+)				
Absolute number (cells/µL)	205.4-893.5	153.7-879.8	468.2	405.2
Relative number (%)	12.8-37.1	11.2-43.0	24.4	22.5
CD4+/CD8+ Ratio	0.8-3.7	0.6-4.4	1.8	2.1
B lymphocytes (CD19+)*				
Absolute number (cells/µL)	87.6-517.9	69.1-498.5	233.2	198.9
Relative number (%)	7.0-21.0	5.3-21.6	12.9	11.0
Natural killer cells (NK)*				
Absolute number (cells/µL)	75.5-405.8	92.8-456.0	190.1	213.6
Relative number (%)	4.3-22.7	4.0-24.1	10.1	11.9

CD: cluster of differentiation. *The sum of observations may differ in some variables due to missing data.

## Discussion

In this study, we established reference intervals for lymphocyte subpopulations using peripheral blood from Brazilian adults, examining potential variations by gender and age groups. Our findings revealed statistically significant differences between genders, with most lymphocyte subtypes, especially B lymphocytes, having significantly higher absolute and relative values in women, while NK cells displayed higher values in men, suggesting the need for different reference intervals for men and women. Statistically significant differences emerged among age groups, with adults showing higher absolute and relative values for cytotoxic T lymphocytes and B lymphocytes and the elderly showing significantly higher absolute and relative values for NK cells. However, statistical tests indicated that partitioning by age groups was unnecessary.

Significant variations in T lymphocytes, B lymphocytes, and NK cell counts have been observed among populations across different global regions, underscoring the necessity of establishing distinct reference intervals tailored to specific geographical areas (14-17). Table 7 presents studies that have defined reference intervals for lymphocytes and their subpopulations in different populations from various regions worldwide.

**Table 7:** Reference intervals for lymphocytes in different countries.

Study	ELSA-MG	Sahmoudi et al. (17)	Mishra et al. (18)	Cumbane et al. (19)	Jentsch-Ullrich et al. (20)	Valiathan et al. (21)
Country	Brazil	Morocco	Nepal	Mozambique	Germany	Florida (USA)
n	344	154	400	419	100	100
Men/women	170/174	64/78	243/157	216/203	50/50	33/67
Average age	58	NI	27	21	43	38
Age intervals	42-82	18-63	18-60	19-24	19-85	21-67
Technology	Sysmex XN-3000/ FACSCalibur	FACSCalibur/ Sysmex SF-3000	FACSCalibur	FACSCalibur	FACSCalibur/ Advia120	FACSCalibur
Total lymphocytes (×10^3^/µL)	1.0-2.9	0.5-4.2	NI	1.0-2.9	1.14-3.38	1.2-4.2
T lymphocytes (CD3+)						
Absolute number (cells/µL)	721.0-2316.3	305.4-2633.8	743-2803	861-2571	780-2240	983-3572
Relative number (%)	59.3-84.5	46.2-85.7	NI	54.0-81.0	53-83	65-88
Helper T lymphocytes (CD3+/CD4+)						
Absolute number (cells/µL)	421.4-1549.6	103.5-1545.1	380-1553	455-1536	490-1640	491-2000
Relative number (%)	32.5-61.6	22.18-58	NI	29.0-53.0	30-59	26-62
Cytotoxic T lymphocytes (CD3+/CD8+)						
Absolute number (Cells/µL)	175.3-893.5	81-1019.9	NI	235-958	170-880	314-2087
Relative number (%)	12.3-39.1	12.3-40.5	NI	14.0-36.0	10-40	14-44
CD4+/CD8+ ratio	0.8-4.2	NI	NI	NI	0.9-5.0	0.6-4.4
B lymphocytes (CD19+)						
Absolute number (cells/µL)	85.2-501.6	34.8-506.3	NI	NI	80-490	64-800
Relative number (%)	5.6-21.0	4.38-29	NI	NI	5-21	2-27
Natural killer cells (NK)						
Absolute number (cells/µL)	83.9-423.4	34.8-506.3	NI	NI	80-690	27-693
Relative number (%)	4.3-23.5	1.5-28.5	NI	NI	5-32	2-27

NI: not informed.

Upon comparing our total reference population results with those of other studies, we noticed that the values of total lymphocytes align with those found in Mozambique and differed from those of other studies (17,18). T lymphocyte levels were lower than those found in Morocco (14), Nepal (18), and Mozambique (19). Cytotoxic T lymphocytes and B lymphocytes were similar to those reported in Germany, while NK cells exhibited a higher minimum limit compared to the German (20) and Moroccan (17) findings. Furthermore, the reference intervals identified in this study deviated from those observed in other regions of the Americas, such as South Florida (21), Peru (22), and Cuba (15).

In comparison with other Brazilian studies, our study showed lower counts of total lymphocytes, T lymphocytes, helper T lymphocytes, and cytotoxic T lymphocytes (Table 8) (23-25). Nevertheless, the comparison should be interpreted with caution due to the diverse regional, age, and other demographic characteristics of the analyzed populations. Similar regional differences were also reported in India (26), Nepal (18), and China (27), which could be attributed to biological factors like age, gender, and ethnicity (28,29), as well as lifestyle factors such as alcohol consumption, smoking, diet, and stress levels (26,30,31). Environmental elements, such as exposure to infectious diseases and socioeconomic factors, might also contribute (17).

**Table 8:** Comparison of T lymphocyte subsets between populations from regions of Brazil.

Study	ELSA-MG	Torres et al. 2013 (8)	Morais et al. 2023 (25)	Rudolf-Oliveira et al. 2015 (24)
Regions of Brazil	Southeast	North, Northeast, Midwest, Southeast, and South	Midwest	South
n	344	641	115	238
Men/women	170/174	403/238	NI	134/104
Average age	58	33	38	27.3
Age intervals	42-82	19-56	18-63	16-56
Technology	Sysmex XN-3000/ FACSCalibur	FACSCalibur	FACSCalibur	Sysmex XE 2100D/FACSCanto II
Total lymphocytes	1000-2900	1257-4104	NI	NI
T lymphocytes (CD3+)				
Absolute number (cells/µL)	721.0-2316.3	1025-2904	NI	718-2494
Relative number (%)	59.3-84.5	NI	48.7-77.2	51.3-83.5
Helper T lymphocytes (CD3+/CD4+)				
Absolute number (cells/µL)	421.4-1549.6	540-1731	NI	456 -1492
Relative number (%)	32.5-61.6	NI	18.2-49.8	24.4-54.2
Cytotoxic T lymphocytes (CD3+/CD8+)				
Absolute number (cells/µL)	175.3-893.5	263-1189	NI	272-1144
Relative number (%)	12.3-39.1	NI	12.6-34.3	12.8-40.2
CD4+/CD8+ ratio	0.8-4.2	NI	NI	0.68-3.61
B lymphocytes (CD19+)				
Absolute number (cells/µL)	85.2-501.6	NI	NI	110-618
Relative number (%)	5.6-21.0	NI	5.4-17.7	6.3-20.8
Natural killer cells (NK)				
Absolute number (cells/µL)	83.9-423.4	NI	NI	82-760
Relative number (%)	4.3-23.5	NI	2.4-18.9	3.7-28.5

NI: not informed.

T, helper T, and B lymphocytes were higher but NK cells were lower in women than in men. A comparable pattern was observed in another study involving individuals from southern Brazil, where women exhibited significantly higher counts of T lymphocytes, helper T cells, and cytotoxic T cells, while men had significantly higher counts of NK cells (24). Elevated counts of these cells in women were also observed in studies conducted in various countries, including Oman (32), Israel (33), Spain (34), and Nepal (18). However, contrasting patterns were observed in other studies. For instance, a study in Iran revealed a higher percentage of T and helper T lymphocytes in females and a higher percentage of B lymphocytes in men (35). Similarly, a Chinese study reported higher counts of NK cells in men (27).

These variations, alongside ethnic differences, may be due to biological factors. Female sex hormones like estrogen and progesterone, as well as male sex hormones such as testosterone and other androgens, interact with nuclear hormone receptors in several cell types, including immune cells. This interaction modulates a wide array of biological processes, affecting various aspects of the immune system's function (36).

Differences in lymphocyte subpopulations were also found between the adult and elderly groups. Both the absolute and relative values of cytotoxic T lymphocytes and B lymphocytes were notably higher in adults, whereas NK cells were significantly elevated in the elderly. Similarly, a Chinese study indicated a decrease in the absolute count of T, helper T, and cytotoxic T lymphocytes with aging (37). Numerous studies have highlighted the correlation between age and lymphocyte subpopulations (38). A significant decrease in total and B lymphocytes was found in men aged ≥50 years, whereas women aged ≥50 years had a reduction in cytotoxic T lymphocytes (32). Criado et al. (39) documented a gradual rise in NK cell counts during adulthood, especially in women, and noted a gradual decline in B lymphocytes among the elderly.

Although variations were observed, there was no detected need to establish separate reference intervals for lymphocyte subpopulations across the assessed age groups, possibly because of the narrow age fluctuation of the adult group (42-59 years) compared to other studies.

The present study had both limitations and strengths. Among its strengths were the large sample size encompassing diverse sociodemographic characteristics, the inclusion of many variables, and the highly standardized assessment of T lymphocyte, B lymphocyte, and NK cell subpopulations. Notably, the study's main strength lied in its reference population derived from a longitudinal study sample. This differs from existing studies that often include blood donors, who are a group of exceptionally healthy individuals that do not fully reflect the general Brazilian population. However, our study had limitations related to the age of the population, as it did not include young individuals. Furthermore, caution must be exercised when generalizing the findings to the general Brazilian population, given that the ELSA-Brasil consists of a cohort of public civil servants from urban settings and the analytical sample is limited to a single Brazilian state.

The outcomes from this study underscore the necessity of establishing reference intervals tailored to specific populations. Furthermore, the significant differences in the relative and absolute values of certain lymphocyte subpopulations between genders indicate that separate reference intervals for men and women, especially helper T lymphocytes, B lymphocytes, and NK cells, should be considered.
